# Evaluation of Parental Nasomaxillary Asymmetry as a Risk Factor for Development of Palatal Clefts in their Offsprings

**DOI:** 10.5005/jp-journals-10005-1072

**Published:** 2010-09-15

**Authors:** Dinesh Kumar S, K Gopalkrishnan, C Bhasker Rao, Sanjay V Ganeshkar

**Affiliations:** 1 Assistant Professor, Mahatma Gandhi Dental College and Hospital, Jaipur, Rajasthan, India; 2 Professor, Department of Oral and Maxillofacial Surgery, SDM College of Dental Sciences and Hospital, Dharwad Karnataka, India; 3 Principal, Professor and Head, Department of Oral and Maxillofacial Surgery, SDM College of Dental Sciences and Hospital Dharwad, Karnataka, India; 4 Professor and Head, Department of Orthodontics and Dentofacial Orthopedics, SDM College of Dental Sciences and Hospital Dharwad, Karnataka, India

**Keywords:** Nasomaxillary asymmetry, Orofacial clefting, PA cephalometric analysis, Craniofacial form.

## Abstract

**Background and Objectives :** It has been suggested previously that increased width of midfacial structure is associated with the development of palatal clefting. One of the most important heritable characteristics predisposing towards the development of orofacial clefting in an embryo is craniofacial morphology. The aim of the study was to compare nasomaxillary width of parents of children with unilateral complete cleft lip alveolus and palate with parents of noncleft children.

**Methods :** 25 biologic parent sets of children with unilateral complete cleft lip alveolus and palate and 25 biologic parents of noncleft children were included in this study for PA cephalometric analysis.

**Results :** There was no statistically significant difference between study and control groups. An association was found between the side of the cleft in the affected children and the parents in the same side with narrower nasomaxillary width.

**Interpretation and conclusion :** The result of this study was in contrast with other previous studies. We observed a narrower nasomaxillary width, which suggested that this feature may be of morphogenetic importance in the etiopathogenesis of orofacial clefting in this geographic and ethnic group.

## INTRODUCTION

Etiologic heterogenicity (polygenic and multifactorial) is now the accepted theory in the etiopathogenesis of orofacial clefting (OFC) with contribution from both genetic and environmental sources. Perhaps one of the most important heritable characteristics predisposing towards the development of OFC in an embryo is the craniofacial morphology (Fraser and Pashayan, 1970)^1^.

Fully defining the parental craniofacial morphology in OFC will aid both the identification of the OFC morphogenes and the detection and counseling of parents determined to be at risk of having more children with OFC.

Moreover, the identification of microform features in the relatives of the subjects with OFC, including cranio-facial form, lip pits and nasal deformities will also assert in the elucidation of gene-gene and gene environment interactions.

Transverse asymmetry of the facial and nasomaxillary skeleton is commonly present in individuals with unilateral complete cleft lip alveolus and palate (UCLP) with the nasomaxillary complex being more asymmetric in affected individuals than noncleft controls.

Interestingly, nasomaxillary asymmetry is also present in the general population as previously demonstrated by various investigators employing frontal (posteroanterior) cephalometric radiographs. Furthermore, the parents of children with cleft lip and palate also display asymmetric craniofacial features when compared with parents of noncleft children. Children born with clefts of the lip and palate have disruption of the hard tissues of the nasomaxillary skeleton.

Asymmetries in the nasomaxillary complex are very common in patient with unilateral complete cleft lip alveolus and palate and have been previously studied by means of posteroanterior radiographs.

Although a number of cephalometric studies have identified morphological differences between the parents of children with OFC and comparison groups, no study has investigated craniofacial asymmetry perse as a heritable predisposing factor towards the development of OFC in their offsprings. Specifically, the localization and quantification of craniofacial asymmetry could prove to be a crucial significant research for the morphogenes involved in OFC.

Some clefts are caused by single mutant genes, some are due to chromosomal aberrations, and some are caused by specific environmental agents. The great majority are caused by the interaction of genetic and environmental factors each with relatively small effect.

Many investigators inferred that if facial shape is genetically determined and also related to predisposing the cleft anomaly, the parents of children with cleft lip/palate should have facial dimensions different from those of general population.

The identification of the parental craniofacial form in the etiopathogenesis of OFC may be important for several reasons:

1. The parental craniofacial form (the phenotype) represents the hereditary influences on the craniofacial form of their offspring (the genotype). The craniofacial form in orofacial cleft is considered to be a predisposing factor in the development of OFC. For example, increased head and facial widths would logically mitigate against the palatal shelves for making contact (Fraser and Pashayan, 1970)^1^.
2. The identification of microform features in the relatives of subjects with OFC (e.g. craniofacial form) will assert in the elucidation of the interaction of genes, both with other genes and their products, and with environmental factors.
3. The identification of craniofacial features that are similar in several biological relationships (features that may not seem directly related to the etiopathogenesis of OFC, e.g. dental or auricular anomalies) may assert in the identification of genes involved in the etiopathogenesis of OFC.

Hence, the current study was designed to evaluate the parental nasomaxillary asymmetry as a risk factor for development of palatal clefts in their offsprings.

## METHODOLOGY

The subjects for the study were 25 sets of parents (25 biologic mothers and 25 biologic fathers) of children with unilateral complete cleft lip alveolus and palate. The study group consisted of parents of siblings reporting with unilateral complete cleft lip alveolus and palate deformities to the Dept. of Maxillofacial Surgery and Research Center, SDM College of Dental Sciences and Hospital, Dharwad with an average age of 27 for males and 24 for females.

The affected children included 12 males and 13 females suffering from nonsyndromic unilateral complete cleft lip alveolus and palate. 68% (n = 17) of affected children had left side cleft and 32% (n = 8) had right side cleft.

There were no subjects with syndromic cleft based on family and patient history as well as clinical examination.

17 patients had unilateral complete cleft lip alveolus and palate on the left side of which 9 were males and 8 were females.

8 patients had unilateral complete cleft lip alveolus and palate on the left side of which 3 were males and 5 were females.

The 25 sets of parents constituting the study group had no evidence of any type of cleft while their progenies exhibited unilateral complete cleft lip alveolus and palate. 9 parents sets in the study group had a history of consanguinity.

The control group subjects for comparison with study group were 25 sets of parents (25 biologic mothers and 25 biologic fathers) of noncleft children. The control group consisted of parents of noncleft children visiting for routine dental treatment to the Dept. of Pedodontics, SDM College of Dental Sciences and Hospital, Dharwad with an average age of 28 for males and 25 for females. Subjects for both the groups were Indian nationals.

The criteria of selection for the control group were:

- Parents whose children had no orofacial clefts, no anomaly of skeletal, genetic, endocrinal or any other nature.
- Subjects with no gross skeletal defects. Although malocclusion was accepted.
- A full compliment of teeth from second molar to second molar in both jaws.
- Individuals who had no diseases of skeletal genetic or endocrine nature.

### Procedure for Obtaining PA Radiograph

**Fig. 1:**
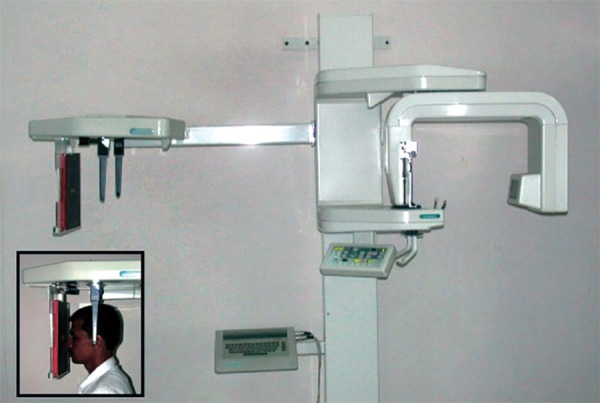
Planemecca PM 2002 CC Proline Panoramic X-ray unit (Inset-picture of standard patient position)

**Fig. 2:**
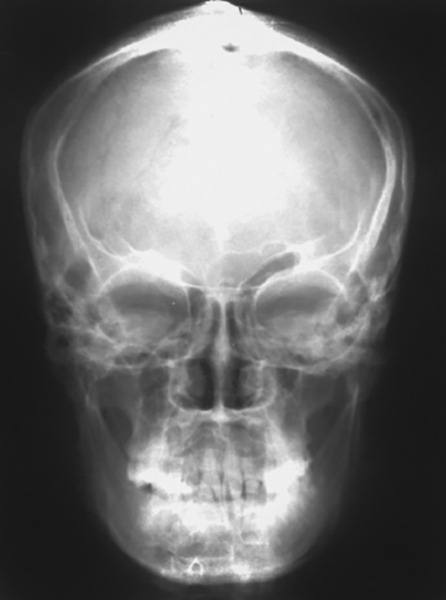
Posteroanterior (frontal) cephalometric radiograph

A total of 100 posteroanterior (frontal) cephalometric radiographs were obtained using a standard technique on a Planemecca PM 2002 CC Proline Panoramic X-ray unit within a period of 6 months (Fig. 1 and 2). Ecta speed Kodak diagnostic films of size 8” × 10” were used. The tube voltage was kept at 80 kV, tube current at 12 mA and exposure time at 2.5 seconds.

Following the standard technique, the head was stabilized in the cephalostat with the help of ear rods (Fig. 1). The posteroanterior cephalograms were taken with the teeth in centric occlusion. The head position in the cephalostat was carefully checked so that Frankfort horizontal plane was parallel to the floor. Care was also taken to see that there was no rotation of the head. The distance between the film cassette and the ear rods and between the ear rods and the source of radiation were kept fixed so that the magnification was standardized. The central ray was made to pass through the center of midsagittal plane so that the magnification of right and left sides of the face was same.

Each radiograph (Fig. 2) was traced on a 0.003” acetate matte tracing paper from (Garware) with a 0.3 mm lead pencil. Each tracing was approved by 2 faculty members in the department of orthodontics, so as to minimize observer errors.

**Fig. 3:**
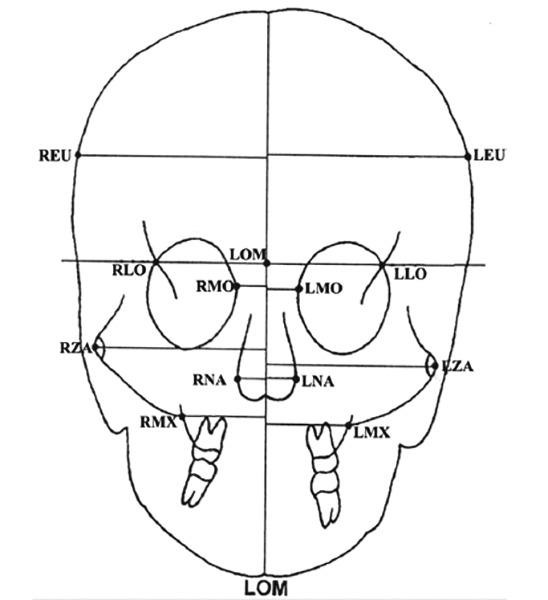
Landmarks, reference lines, and measurements used in the tracings of the posteroanterior cephalometric radiographs

For analysis of these frontal (PA) cephalometric radiographs, five bilateral landmarks were identified and traced on each radiograph and each measurement was assessed (Fig. 3), as demonstrated by Sassouni (1958), Yen (1960), Grummons and Kappeyne (1987)^5^ and Laspos et al (1997)^9^:

1. *Euryon (REU and LEU),* the most lateral point at the parietal surface.
2. *Medioorbitale (RMO andLMO),* the most medial point on the medial orbital margin.
3. *Nasal point (RNA andLNA),* the most lateral point in the nasal cavity.
4. *The maxillary notch (RMX andLMX),* the most medial point on the maxilloalveolar surface.
5. *Zygoma (RZA and LZA),* the most lateral point on the zygomatic arch.

The line connecting latero-orbitale (RLO and LLO) ROL, the intersection between the lateral margin of the orbit and linea innominata, was used as the reference line for vertical measurements.

A line drawn perpendicular to ROL at the midpoint of RLO-LLO was used as the reference line, LOM, for horizontal measurements.

Following measurements of horizontal asymmetry were assessed on the basis of these landmarks (Fig. 3):

1. Head asymmetry, the difference of the perpendicular distance of REU and LEU from LOM.
2. Orbital asymmetry, the difference of perpendicular distance of RMO and LMO from LOM.
3. Nasal asymmetry, the difference of perpendicular difference of the perpendicular distance of RNA and LNA from LOM.
4. Maxillary asymmetry, the difference of perpendicular distance of RMX and LMX from LOM.
5. Zygomatic asymmetry, the difference of perpendicular distance of RZA and LZA from LOM.

One examiner traced and measured all the 100 PA cephalogram.

### Statistical Tests used in the Study

The readings of the 25 study parent sets (25 biologic mother and 25 biologic father) and 25 control parent set (25 biologic mother and 25 biologic father) were subjected to the following statistical tests Mean, Standard Deviation, Student’s unpaired ‘t’ test.

## RESULTS

The ratio of children with left sides unilateral complete cleft lip alveolus and palate versus right side unilateral cleft lip alveolus and palate (UCLAP) was 2:1 in this study.

Table 1 shows details about study subjects. Tables 2 to 5 show cephalometric measurements obtained from both study and control groups. Association of the side of parental asymmetry with side of cleft in their children is summarized in Table 6. Interestingly, of all the facial and nasomaxillary parental structure examined, only nasal and maxillary asymmetry appeared to have any significant association with offspring clefting.

The side of increased parental nasal and maxillary asymmetry was significantly associated (p < 0.0250 for nasal asymmetry and p < 0.0100 for maxillary asymmetry) with the opposite side of cleft in their children (Table 6). In the majority of parents with children suffering from a left cleft, the nasal and maxillary width (RNA, LNA from LOM and RMX, LMX from LOM) was larger on the right side, compared to the left side.

Similarly, in the majority of parents with children suffering from the right sided cleft, nasal and maxillary width was larger on left compared with the right side.

The association of the linear cephalometric variables of parents of nonsyndromic unilateral complete cleft lip alveolus and palate children (study group) and parents of healthy noncleft children (control group) is presented in Table 7. No significant difference in craniofacial morphology between two groups were mainly expressed in the variables of head, orbital zygomatic, nasal and maxillary asymmetry.

The comparison of left and right side of affected children with their parents is summarized in Table 8. There was a significant p-value for LNA and RMX from LOM.

## DISCUSSION

The aim of the study was to evaluate the parental nasomaxillary asymmetry as a risk factor for development of palatal clefts in their offspring by comparing the nasomaxillary width obtained from PA cephalograms of parents of children with nonsyndromic unilateral complete cleft lip alveolus and palate with parents of noncleft children. The genetic contribution of characteristic craniofacial structure (nasomaxillary asymmetry) in parents of children with nonsyndromic unilateral complete cleft lip alveolus and palate has related to predisposition of non-syndromic unilateral complete cleft lip alveolus and palate in offspring was hypothesized. The focus of this study was to determine differences in craniofacial morphology on PA cephalogram among parents of nonsyndromic unilateral complete cleft lip alveolus and palate children and control group.

**Table 1:** **Table 1:** Study subjects

*Sl.* * No.*		*Sex*		*Age of* *the* * father*		*Age of* *the* *mother*		*Cleft* * side* * UCLAP*		*H/O of* * Consanguinity* * Yes/No*		*Other* *congenital* * abnormality*	
1		F		26		21		Left		No		No	
2		M		27		38		Left		No		No	
3		M		27		26		Left		Yes		No	
4		F		22		19		Left		No		No	
5		M		29		28		Right		Yes		No	
6		F		24		21		Left		Yes		No	
7		M		24		23		Left		No		No	
8		F		30		25		Left		No		No	
9		M		40		26		Right		No		No	
10		F		21		20		Right		Yes		No	
11		M		27		23		Right		Yes		No	
12		F		24		22		Left		No		No	
13		F		32		30		Right		No		No	
14		F		26		24		Left		No		No	
15		F		32		30		Right		No		No	
16		M		26		25		Left		No		No	
17		M		26		21		Left		No		No	
18		F		29		26		Left		No		No	
19		F		28		26		Right		No		No	
20		M		24		20		Left		No		No	
21		F		23		20		Left		No		No	
22		M		30		25		Left		Yes		No	
23		F		28		27		Right		Yes		No	
24		M		30		26		Left		Yes		No	
25		M		24		28		Left		Yes		No	

**Table 2:** **Table 2:** Male subjects study group

*Study* *group*		*Cleft* * side*		*Patient* *sex* * (child)*		*REU*		*LEU*		*RMO*		*LMO*		*RZA*		*LZA*		*RNA*		*LNA*		*RMX*		*LMX*	
SP 1		L		F		6.9		7.5		1.6		1.6		6.9		7.1		1.7		1.5		3.3		3.3	
SP 2		L		M		7.2		7.4		1.2		1.1		7		6.9		2		1.5		3.4		2.9	
SP 3		L		M		7.3		6.8		1.2		1.3		6.8		6.5		1.8		1.9		3.4		3.2	
SP 4		L		F		7.1		6.9		1.5		1.4		7		6.8		1.8		1.7		3.4		3.1	
SP 5		R		M		8.3		6.8		1.3		1.3		7.3		6.2		1.8		2		3		3.2	
SP 6		L		F		7.2		7		1.6		1.7		6.7		6.4		1.8		1.6		3.4		3.1	
SP 7		L		M		7.1		7.7		1.1		1.3		6.4		6.9		1.5		1.8		3.2		3.5	
SP 8		L		F		6.5		6.7		1.1		1.4		7		6.8		1.6		1.6		3.4		3.1	
SP 9		R		M		6.6		7.1		1.5		1.4		6.5		6.6		2		2		3.4		3.7	
SP 10		R		F		7.1		6.9		1.6		1.5		7.1		6.8		1.8		1.7		3.4		3.1	
SP 11		R		M		6.9		6.8		1.1		1.3		6.6		7.1		1.4		2		3.2		3.8	
SP 12		L		F		7.1		7.3		1.4		1.2		6.9		6.5		1.8		1.3		3.6		3.1	
SP 13		R		F		6.7		6.4		1.3		1.3		6.6		6.6		1.4		1.7		2.8		3	
SP 14		L		F		7		6.6		1.2		1.2		6.6		5.9		1.9		1.1		3.2		2.5	
SP 15		R		F		7.3		7.2		1.3		1.5		6.9		7		1.6		1.6		3		2.6	
SP 16		L		M		7.3		6.6		1.3		1.1		7.2		6.6		2		1.6		3.5		3.1	
SP 17		L		M		7.2		7		1.4		1.6		7		7.1		2.1		1.6		3.8		3.3	
SP 18		L		F		7.5		7		1.6		1.6		7.4		6.9		2.3		1.6		3.8		3.2	
SP 19		R		F		7.7		7.5		1.4		1.3		7.1		6.6		1.8		1.2		3.5		2.8	
SP 20		L		M		7.7		7.7		1.5		1.6		7.1		7.2		1.9		1.7		3.7		3.5	
SP 21		L		F		7		7.3		1.3		1.2		6.9		6.9		1.6		1.6		3.2		3.2	
SP 22		L		M		7.5		7.4		1.4		1.8		7.1		7.2		1.3		1.5		3.2		3.2	
SP 23		R		F		7.1		6.8		1.3		1.2		6.5		6.3		1.6		1.7		3.1		3.2	
SP 24		L		M		7		7.4		1.6		1.6		6.7		6.7		1.8		1.7		3.6		3.6	
SP 25		L		M		8.4		7.3		1.4		1.4		7.4		6.7		2		1.3		3.7		3.1	

**Table 3:** **Table 3:** Female subjects study group

		*Cleft* *side*		*Patient* * sex* *(child)*		*REU*		*LEU*		*RMO*		*LMO*		*RZA*		*LZA*		*RNA*		*LNA*		*RMX*		*LMX*	
SP1		L		F		6.8		7.3		1.3		1.4		6.5		6.5		1.8		1.7		3.5		3.3	
SP2		L		M		7.5		7.4		1.6		1.5		6.7		6.4		1.6		1.2		3.4		2.8	
SP3		L		M		6.8		6.8		1.3		1.3		6.1		6.1		1.3		1.1		3.1		2.7	
SP4		L		F		6.8		6.4		1.6		1.5		6.4		6.2		1.7		1.7		2.9		3.3	
SP5		R		M		6.7		6.5		1.4		1.2		6.3		6.5		1.9		1.8		3.1		3.4	
SP6		L		F		7		6.7		1.5		1.5		7.4		6.8		2		1.4		3.8		3.1	
SP7		L		M		7.5		6.6		1.7		1.5		6.9		5.8		2.1		1.3		3.5		2.6	
SP8		L		F		6.8		6.9		1.2		1.4		6.5		6.4		1.6		1.6		3.3		3.2	
SP9		R		M		6.7		6.7		1.5		1.2		6.5		6.6		1.8		1.9		3.5		3.7	
SP10		R		F		6.8		6.5		1.4		1.6		6.6		6.1		1.9		1.6		3.7		2.9	
SP11		R		M		6.9		6.9		1.3		1.2		6.7		6.3		1.8		1.4		3.4		3	
SP12		L		F		7.1		6.9		1.6		1.3		6.7		6.4		1.8		1.2		3.5		3.2	
SP13		R		F		6.8		6.8		1.1		1.2		6.1		5.9		1.6		1.7		2.7		2.9	
SP14		L		F		7		6.4		1.3		1.1		6.3		6.3		2		1.6		3.2		3.1	
SP15		R		F		7.3		6.3		1.5		1.2		6.4		5.9		1.8		1.8		2.9		2.9	
SP16		L		M		7.6		7.3		1.8		1.5		7		6.5		1.5		1.3		3.3		3	
SP17		L		M		8.2		6.4		1.6		1.3		8.1		6.1		2.1		1.1		3.6		2.9	
SP18		L		F		7.1		6.8		1.1		1		6.2		6.1		1.6		1.3		3.4		3.1	
SP19		R		F		7		6.8		1.2		1.3		6		5.8		1.6		1.3		2.9		2.6	
SP20		L		M		6.9		6.9		1		1.2		6.5		6.3		1.8		1.5		3		2.8	
SP21		L		F		7.1		7		1.3		1.4		6.9		6.8		1.6		1.5		3.4		3.5	
SP22		L		M		6.2		7		1.4		1.2		6.2		6		1.5		1.2		3.2		2.7	
SP23		R		F		6.8		6.7		1.5		1.5		6.6		6.4		1.7		1.7		3.2		3.2	
SP24		L		M		7.9		7.6		1.4		1.5		6.7		6.6		1.7		1.8		3.4		3.6	
SP25		L		M		7		6.7		1.3		1.2		6.7		6.2		1.9		1.3		3.3		2.7	

**Table 4:** **Table 4:** Male subjects control group

		*REU*		*LEU*		*RMO*		*LMO*		*RZA*		*LZA*		*RNA*		*LNA*		*RMX*		*LMX*	
CP1		7		6.5		1.5		1.4		6.7		6.6		2		1.9		3.4		3.4	
CP2		7.5		6.9		1.4		1.5		6.8		6.5		1.9		1.6		3.1		2.9	
CP3		6.8		6.2		1.1		1.1		6.5		6.5		1.7		1.6		3.1		3	
CP4		7.4		6.6		1.6		1.6		6.7		7		1.8		1.8		3.4		3.3	
CP5		7.3		7		1.1		1.1		7		6.8		1.5		1.5		3.5		3.3	
CP6		7.1		7.2		1.4		1.4		7		7		1.8		1.5		3.5		3.4	
CP7		7.4		6.7		1.4		1.2		6.9		6.9		2.2		2		3		3	
CP8		7.1		7.2		1.5		1.5		7.2		7		1.7		1.7		3.4		3.4	
CP9		7.7		6		1.5		1.2		6.9		6.4		1.7		1.6		3.2		3.2	
CP10		6.9		6.8		1.1		1.3		6.8		6.5		1.8		1.4		3.6		3.2	
CP11		6.7		6.7		1.4		1.4		6.8		6.7		1.5		1.5		3.2		3.1	
CP12		7.7		6.5		1.2		1.2		7.3		6.3		2.2		1.8		3.8		3.2	
CP13		7.3		6.1		1.3		1.2		6.9		6.6		1.9		1.5		3.5		3.2	
CP14		7.3		7.3		1.5		1.5		7		7.4		1.3		1.8		2.8		3.5	
CP15		7.2		7		1.5		1.4		7.1		7.1		1.7		1.6		3.3		3.2	
CP16		7.3		6.7		1.5		1.6		7.1		7		1.9		1.5		3.5		3.2	
CP17		7.1		6		1.5		1.3		6.7		6.3		1.5		1.3		3.1		2.9	
CP18		7		6.6		1.6		1.4		7		6.7		2		1.1		3.1		3	
CP19		7		6.6		1.3		1.2		7.1		6.8		1.9		1.6		3.3		3.2	
CP20		7.2		6.9		1.4		1.4		6.8		6.5		1.7		1.3		3.1		3.1	
CP21		7.2		6.5		1.2		1.3		7.4		6.5		1.8		1.3		3.6		3.2	
CP22		7.1		7.3		1.1		1.1		6.9		6.7		1.5		1.4		3.2		3	
CP23		7		6.5		1.6		1.5		6.8		6.5		1.7		1.2		3.3		3.1	
CP24		8.3		6.2		1.1		1.2		7.5		6.6		1.9		1.2		3.6		2.9	
CP25		7.3		6.2		1.3		1.3		6.8		6.3		2		1.5		3		3.1	

**Table 5:** **Table 5:** Female subjects control group

		*REU*		*LEU*		*RMO*		*LMO*		*RZA*		*LZA*		*RNA*		*LNA*		*RMX*		*LMX*	
CP1		7.3		6.9		1.1		1.1		6.5		6.2		2.1		1.8		3.1		3.1	
CP2		6.2		6.3		1.4		1		6.2		6		1.7		1.3		3.4		3	
CP3		6.9		6.5		1.3		1		6.6		6.1		1.8		1.3		3.3		2.6	
CP4		6.5		6.5		1.2		1.2		6.1		6		1.6		1.4		3.3		3.1	
CP5		7.1		6.5		1.4		1.4		6.9		6.6		1.7		1.7		3.4		3.2	
CP6		7		7		1.5		1.5		6.9		6.8		2.1		2.1		3.5		3.4	
CP7		7.3		6.7		1.5		1.2		6.9		6.8		1.7		1.7		3.4		3.2	
CP8		6.4		6.4		1.3		1.4		6.5		6.7		1.7		1.7		3.3		3.3	
CP9		7.1		6.2		1.3		1.2		6.7		6.2		2		1.6		3.3		3	
CP10		6.3		6.5		1.2		1.2		6.5		6.3		1.7		1.3		3.3		2.9	
CP11		6.7		6.1		1.3		1		6.3		6		1.8		1.6		3.3		3	
CP12		7.1		6.2		1		1		6.6		6.1		1.7		1.3		3.4		3	
CP13		7.5		6.8		1.2		1.3		7.2		6.8		2		1.4		3.5		3.1	
CP14		7.1		7.2		1.4		1.2		6.5		6.3		1.6		1.4		3.3		2.8	
CP15		8.2		6.2		1.4		1.3		7.4		6.4		1.8		1.7		3.5		3.1	
CP16		7		7.2		1.3		1.4		6.6		6.4		1.8		1.2		3.1		3.2	
CP17		7		6.6		1.4		1.4		6.4		6.6		1.5		1.7		3		3.1	
CP18		7.7		6.8		1.5		1.4		6.7		6.4		1.8		1.8		3.2		3.2	
CP19		7		6.7		1.4		1.2		6.6		6.5		1.7		1.7		3.4		3.4	
CP20		7.1		6.5		1.2		1.1		6.6		6.6		1.5		1.5		3		3	
CP21		6.5		6.9		1.4		1.5		6		6.2		1.7		1.4		3.2		3	
CP22		6.2		6.6		1.2		1.1		6		6.2		1.6		1.5		3.1		3.1	
CP23		7.4		7		1.4		1.4		6.6		6.6		1.5		1.4		3.2		3.1	
CP24		6.3		6.7		1.2		1.1		6.2		6.6		1.4		1.7		3		3.3	
CP25		7		6.3		1.3		1.3		6.9		6.4		2		1.5		3.2		3.1	

**Table 6:** **Table 6:** Association of the side of parental asymmetry with side of cleft in their children

*Variables*		*Group*		*Rt > Lt*		*Rt < Lt*		*Rt = Lt*		*Total*		*Chi-square*		*P-value*	
		Left		21		10		3		34					
Head asymmetry		Right		12		1		3		16		3.8352		0.1470	
		Grand total		33		11		6		50					
		Left		16		11		7		34					
Orbital asymetry		Right		8		5		3		16		0.0421		0.9792	
		Grand total		24		16		10		50					
		Left		24		5		5		34					
Zygomatic asymmetry		Right		10		5		1		16		2.2419		0.3260	
		Grand total		34		10		6		50					
		Left		26		4		4		34					
Nasal asymmetry		Right		6		6		4		16		7.3759		0.0250	
		Grand total		32		10		8		50					
		Left		26		4		4		34					
Maxillary asymmetry		Right		6		8		2		16		9.2142		0.0100	
		Grand total		32		12		6		50					

**Table 7:** **Table 7:** Association of the study and control group of parental asymmetry

*Variables*		*Group*		*Rt > Lt*		*Rt < Lt*		*Rt = Lt*		*Total*		*Chisquare*		*P-value*	
		Control		36		9		5		50					
Head asymmetry		Study		33		11		6		50		0.4210		0.8100	
		Grand total		69		20		11		100					
		Control		21		9		20		50					
Orbital asymetry		Study		24		16		10		50		5.4933		0.0642	
		Grand total		45		25		30		100					
		Control		37		7		6		50					
Zygomatic asymmetry		Study		34		10		6		50		0.6562		0.7203	
		Grand total		71		17		12		100					
		Control		36		3		11		50					
Nasal asymmetry		Study		32		10		8		50		4.4782		0.1066	
		Grand total		68		13		19		100					
		Control		34		5		11		50					
Maxillary asymmetry		Study		32		12		6		50		4.4135		0.1101	
		Grand total		66		17		17		100					

**Table 8:** **Table 8:** Comparison of left and right side of the cleft children with study group

*Variables*		*Left*				*Right*				*t-value*		*P-value*		*Signi.*	
		*Mean*		*Std.Dev.*		*Mean*		*Std.Dev.*							
REU		7.1853		0.4377		7.0438		0.4412		1.0640		0.2927		NS	
LEU		7.0206		0.3756		6.7938		0.3021		2.1119		0.0399		S	
RMO		1.3941		0.1953		1.3563		0.1459		0.6888		0.4942		NS	
LMO		1.3794		0.1919		1.3250		0.1342		1.0202		0.3128		NS	
RZA		6.8206		0.4081		6.6125		0.3519		1.7537		0.0859		NS	
LZA		6.5471		0.3735		6.4188		0.3816		1.1253		0.2660		NS	
RNA		1.7794		0.2320		1.7188		0.1721		0.9304		0.3568		NS	
LNA		1.4824		0.2208		1.6938		0.2380		-3.0809		0.0034		S	
RMX		3.4000		0.2202		3.1750		0.2864		3.0565		0.0037		S	
LMX		3.1059		0.2785		3.1250		0.3697		-0.2035		0.8396		NS	

The results from our study showed that the parental craniofacial morphology in nonsyndromic unilateral complete cleft lip alveolus and palate statistically does not differ from that of control group and association of side of parental asymmetry with the side of cleft in their children showed that majority of parents with children suffering from cleft in the left side, the nasal and maxillary width was larger on the right side compared to the left side, i.e. children with cleft in left side had small nasal and maxillary width in ipsilateral side of their parents.

Because there is no association between the control and study group, it does not exclude that the importance of craniofacial form has a genetic etiologic factor in the genesis of clefting. Various other studies is in contrast with our study [Nakasima A et al (1983)^4^, Suzuki A et al (1991)^6^, Raghavan R et al (1994)^7^, Mossey PA et al (1997)^9^, Laspos CP et al (1997)^9^, Mossey PA (1999)^10^, AL Emran SE et al (1999)^11^, Yoon YJ et al (2003)^13^], they found significant association between study and control groups of parental asymmetry and suggested a possible role of craniofacial form in orofacial clefting.

However, although nasomaxillary asymmetry is also present in general population, as previously demonstrated by various investigators employing frontal (posteroanterior) cephalometric radiographs, there are conflicting reports as to which side of the craniofacial skeleton is dominant in the general population. Shah AM et al (1978)^3^ in a frontal cephalometric study on 18 to 25-year-old subjects reported that the craniofacial skeleton was found asymmetric in the general population with the right side being greater than the left. In contrast, Vig PS etal (1975)^2^ reported craniofacial asymmetry with the left side being overall greater than the right.

Association of the side of parental asymmetry with the side of cleft in their children showed that majority of parents with children suffering from unilateral complete cleft lip alveolus and palate on the left side, the nasal and maxillary width was larger on the right side compared to the left side, i.e. children with cleft in left side had small nasal and maxillary width on the same side of their parents.

Curiously, the results from our study were not in complete agreement with many previous studies. In particular, our finding of a smaller nasal and maxillary width in the ipsilateral side of the parents contrasted with an increased nasal and maxillary width found in Suzuki A et al (1991)^6^, Raghavan R et al (1994)^7^, AL Emran SE et al (1999)^11^, and Yoon YJ et al (2003)^13^. This could be due to morphogenetically distinct study sample as compared to other geographic areas and ethnic grouping. GT Mclntyre et al (2003)^12^ found a smaller nasomaxillary width in contrast to other studies. But this study indicated that these features may be of morphogenetic importance in the etiopathogenesis of OFC in this ethnic group and also concluded that craniofacial morphology in OFC differs significantly from the noncleft population.

Some studies did not compare the study group with control group but found ipsilateral increase in the nasomaxillary width in the parents as one of the possible causes for development of unilateral complete cleft lip alveolus and palate [Yoon YJ et al (2003)^13^].

Our study compared only parents of complete cleft lip alveolus and palate with control group. There were no quantitative difference of craniofacial structure identified in parents of nonsyndromic unilateral complete cleft lip alveolus and palate (study group) in this investigation as compared to the parents of noncleft children (control group). This study was unable to associate craniofacial form of parents with unilateral complete cleft lip alveolus and palate children. This does not indicate that such predisposing facial structures are unlikely to be only the determinant of cleft susceptibility. Further studies are required in this field, which might include large appropriate sample size.

9 parent sets in study group gave the history of consanguinity. This adds to the etiologic heterogenicity of orofacial clefting in our study group. This consanguinity between the parents could be stronger feature compared to the craniofacial form for the development of orofacial clefting in their offsprings.

This investigation suggested that unilaterally decreased nasomaxillary width in parents may play as a risk factor for development of palatal cleft in the offspring in our study group. This study suggested that a systematic approach in selection of subjects in both study and control group can help better understanding of genetic factors of craniofacial form associated with development of unilateral complete cleft lip alveolus and palate. This may ultimately contribute in the assessment of risk for palatal clefting in their offsprings. The features of this study was in contrast with many other previous studies suggesting that this feature may be of morphogenetic importance in the etiopathogenesis of OFC in this geographical and ethnic group.
